# Synthesis and characterisation of DOTA‐kisspeptin‐10 as a potential gallium‐68/lutetium‐177 pan‐tumour radiopharmaceutical

**DOI:** 10.1111/jne.13487

**Published:** 2025-01-07

**Authors:** Janke Kleynhans, Robert Reeve, Cathryn H. S. Driver, Biljana Marjanovic‐Painter, Mike Sathekge, Jan Rijn Zeevaart, Thomas Ebenhan, Robert P. Millar

**Affiliations:** ^1^ NuMeRI, Nuclear Medicine Research Infrastructure NPC Steve Biko Academic Hospital Pretoria South Africa; ^2^ Centre for Neuroendocrinology University of Pretoria Pretoria South Africa; ^3^ The South African Nuclear Energy Corporation (NECSA) Pelindaba South Africa; ^4^ Department of Nuclear Medicine and Steve Biko Academic Hospital University of Pretoria Pretoria South Africa; ^5^ Department of Integrative Biomedical Sciences, Institute of Infectious Diseases and Molecular Medicine University of Cape Town Cape Town South Africa

**Keywords:** antimetastatic, peptide receptor radionuclide therapy (PRRT), pharmacokinetics, positron emission tomography (PET), tumorigenesis

## Abstract

Kisspeptin (KISS1) and its cognate receptor (KISS1R) are implicated in the progression of various cancers. A gallium‐68 labelled kisspeptin‐10 (KP10), the minimal biologically active structure, has potential as a pan‐tumour radiopharmaceutical for the detection of cancers. Furthermore, a lutetium‐177 labelled KP10 could find therapeutic application in treating oncological diseases. DOTA (1,4,7,10‐tetraazacyclododecane‐1,4,7,10‐tetraacetic acid) was attached to the NH2‐terminus of KP10 as we posited from our previous publications that this modification would not impair biological activity. Here, we showed that the biological activity, as monitored by stimulation of inositol phosphate accumulation in HEK293 transfected with the KISS1R gene, was indeed similar for KP10 and DOTA‐KP10. The optimisation of radiolabelling with gallium‐68 and lutetium‐177 is described. Stability in serum, plasma and whole blood was also investigated. Pharmacokinetics and biodistribution were established with micro‐PET/CT (positron emission tomography/computerised tomography) and ex vivo measurements. Dynamic studies with micro‐PET/CT demonstrated that background clearance for the radiopharmaceutical was rapid with a blood half‐life of 18 ± 3 min. DOTA‐KP10 demonstrated preserved functionality at KISS1R and good blood clearance. These results lay the foundation for the further development of DOTA‐KP10 analogues that have high binding affinity along with proteolytic resistance.

## Graphical abstract

DOTA‐kisspeptin‐10, labelled with gallium‐68 and lutetium‐177, is presented for as a possible pan‐tumour radiopharmaceutical. The modified peptide retains biological activity at the KISS1 receptor, demonstrates good stability and exhibits rapid blood clearance, supporting its potential for cancer detection and therapy in future theranostic applications.

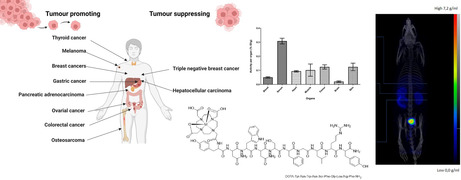

## 1 INTRODUCTION

There is growing evidence that tumorigenesis is influenced by the antimetastatic kisspeptin gene (KISS1 gene)^1^, ^2^, ^3^ that encodes pro‐kisspeptin that is proteolytically cleaved to kisspeptin‐54, kisspeptin‐14, kisspeptin‐13 and kisspeptin‐10. All the cleaved kisspeptins are biologically active and have the same 10 amino acid carboxyl terminus sequence. The KISS1 gene and KISS1 receptor (KISS1R) display variable mRNA expression levels in different cancers and at different cancer stages.^1^ This offers an opportunity to explore the development of a novel kisspeptin theranostic radiopharmaceutical targeting a variety of tumour types.

**FIGURE 1:**
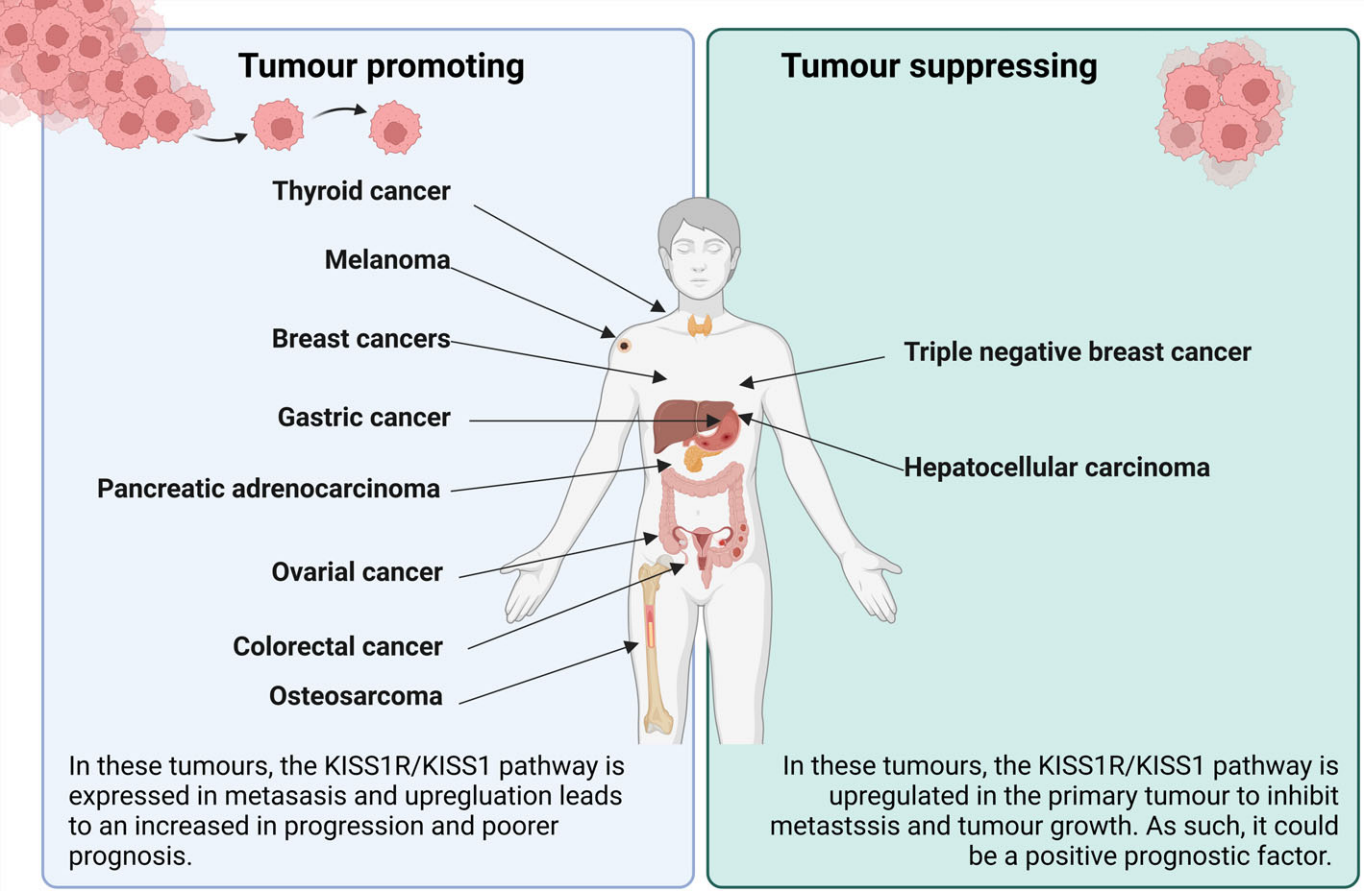
The potential roles of KISS/KISS1R expression in different tumour types (drawn with a licensed version of BioRender.com).^3^, ^5^, ^6^, ^7^, ^8^

A major biologically active product of the kisspeptin precursor is kisspeptin‐10 (KP10).^1^, ^3^, ^4^ KP‐10 together with KISS1R has been observed to display a stimulatory or inhibitory role in tumours depending on the type of cancer. In some tumour types, the KISS1R/KISS1 pathway is upregulated in the primary tumour and serves as a tumorigenesis and metastasis suppressor.^2^, ^3^ Contrarily, in other tumour types, it is widely expressed in metastases and leads to an increase in progression and poorer prognosis.^3^, ^4^ This apparent contradiction/conundrum is also characteristic of other mediators in cancer such as NFKB, c‐MYC, AMP‐activated protein kinase, transforming growth factor β, SKY and hyaluronidase that have been demonstrated to fulfil dual roles.^3^ The application of the KISS1R as a target in the clinical setting therefore appears to depend on the type of primary tumour and KISS1R expression profile.^3^ The potential roles of KISS/KISS1R expression in different tumour types are summarised in Figure 1, updated from the review by Guzman et al.^3^ Radionuclide‐labelled kisspeptin peptides provide a potential target for diagnostic imaging and if proven efficacious, might have application as a pan‐tumour therapeutic radiopharmaceutical (theranostic). A number of cancers have been found to have increased or decreased expression of kisspeptin and its cognate receptor.^3^, ^5^, ^6^, ^7^, ^8^ Although it is unclear what this means in the evolution of the disease and response to therapy, it is speculated that for some cancers expressing these would indicate a poor prognosis and others a good prognosis in view of the antimetastatic properties first described in a melanoma cell line but variable findings in cancers.

In the development of a kisspeptin theranostic agent, the appropriate diagnostic and therapeutic radionuclide along with an applicable chelator needs to be selected. The choice of gallium‐68 as a diagnostic nuclide is logical because of the logistics and convenience associated with its clinical use, as described previously.^9^, ^10^ The combination of a therapeutic radionuclide, lutetium‐177, along with gallium‐68 presents a theranostic partnership as demonstrated by gallium‐68 or lutetium‐177‐labelled PSMA (prostate‐specific membrane antigen) used in clinical practice the staging of prostate cancer and treatment thereof. The optimal chelator choice for these nuclides as is widely implemented in the clinical setting for FDA‐approved radiopharmaceuticals is DOTA (1,4,7,10‐tetraazacyclododecane‐N,N′,N″,N‴‐tetraacetic acid).^11^

As a site for conjugation of DOTA to KP10, we chose the NH2‐terminus as this is extended in the natural kisspeptins and our studies on modification of this site show it is well tolerated^11^, ^12^, ^13^, ^14^ and the addition of DOTA at the NH2‐terminus was unlikely to hinder the key binding/activation residues, Phe6, Arg9 and Phe10. These residues play an important role in accessing the hydrophobic pocket of the KISS1R receptor.^12^ The literature also indicates that Asn4, Ser5, Gly7, Arg9 and Phe10 are involved in the binding and activity of KP10 at the KISS1R^12^, ^14^ but these would not be affected by the NH2 terminus addition of DOTA.

**FIGURE 2:**
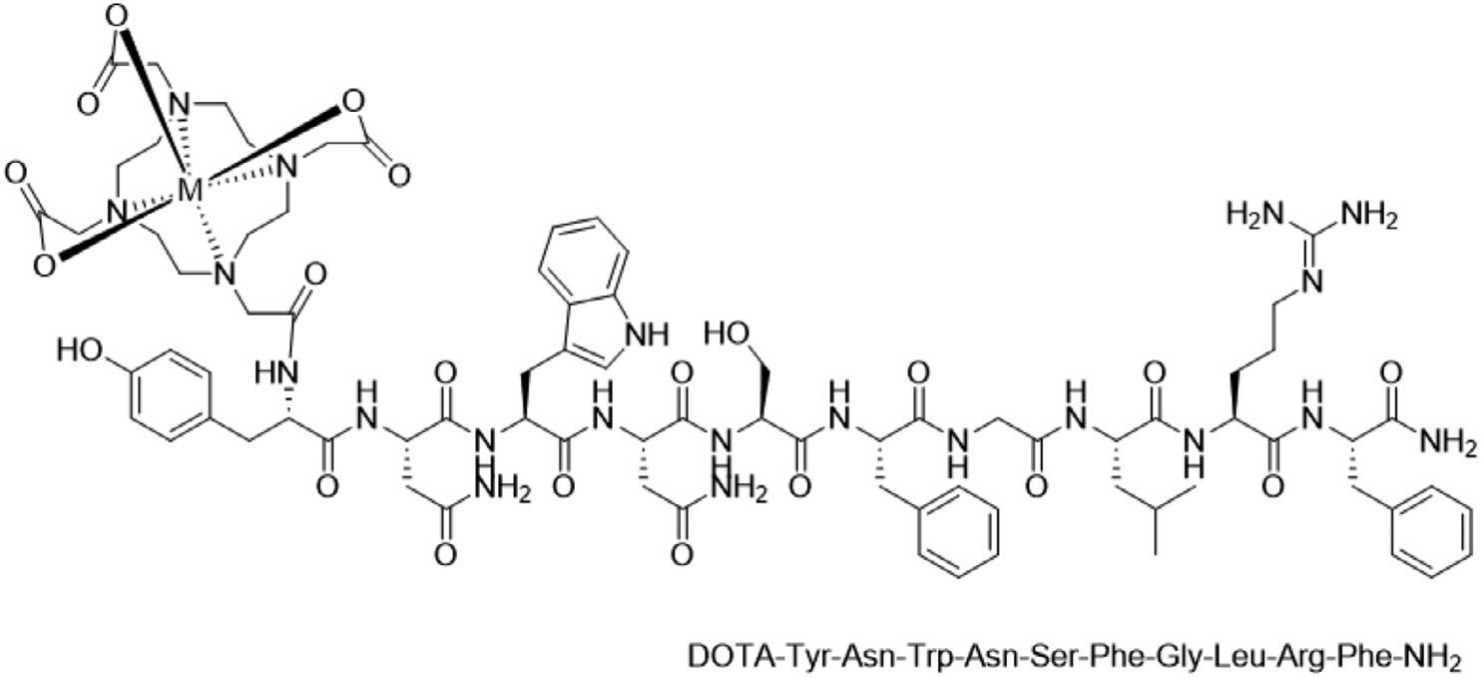
Structure of DOTA‐KP10. M = gallium‐68 or lutetium‐177.

We therefore proposed that [^68^Ga]Ga‐DOTA‐KP10 would be efficacious (Figure 2). As shown for other [^68^Ga]Ga‐DOTA peptides, lutetium‐177 labelling would potentially serve as a therapeutic but also for surgical debulking in primary tumours where KISS1R has a tumour suppression effect. It could also provide [^177^Lu]Lu‐DOTA‐KP10 therapy for breast cancer and hepatocellular carcinoma where metastases express the KISS1R.^3^, ^5^, ^7^

The current study aimed to optimise the radiolabelling of DOTA‐KP10 to produce [^68^Ga]Ga‐DOTA‐KP10 and [^177^Lu]Lu‐DOTA‐KP10 using a simple and repeatable procedure.

## 2 MATERIALS AND METHODS

### 2.1 Materials

The 1850‐MBq germanium‐68 generator was obtained from iThemba LABS (Somerset West, South Africa). Hydrochloric acid 0.6 M for elution was obtained from ABX biopharmaceuticals GmbH (Radeberg, Germany). Lutetium‐177 (n.c.a) was obtained from NTP Radioisotopes SOC Ltd. (Hartebeespoort, South Africa).

DOTA‐KP10 was synthesised by GL Biochem (Shangai, China) by conventional solid phase technology with the attachment of DOTA at the NH2‐terminus of KP10 (Supplementary Data A). The molecular weight by mass spectrometry was the correct 1688.43 g/mol and purity 96%. The structure is shown in Figure 2. All solvents, reagents and materials used during the synthesis and radiochemical analysis (supra‐pure HCl, trifluoroacetic acid, ethanol, acetonitrile, sodium acetate trihydrate, sodium citrate, pH test strips and Millex GV 0.22 μm sterile filters) were purchased from Sigma‐Aldrich (Darmstadt, Germany) or Merck (Kenilworth, New Jersey, USA). Deionised, ultrapure water was produced with a Simplicity 185 Millipore system (Cambridge, USA). The C18 SepPak cartridges for post‐purification were available from Waters (Milford, USA). Phosphate‐buffered saline from ABX (Radeberg, Germany) was used for the final formulation. Materials for the cell culture assay were XtremeGene HP DNA transfection reagent from Roche Diagnostics (Basel, Switzerland), Dulbecco's Modified Eagle Medium (DMEM), high glucose, GlutaMAX™ from Thermo Fisher Scientific (Waltham, USA) and trypsin–EDTA solution from Sigma Aldrich (Sigma‐Aldrich, St Louis, MO, USA). Cell culture plates and other flasks were obtained from Corning Life Sciences (New York, USA). Human embryonic kidney (HEK) 293‐T cells were obtained from ATCC (Manassas, VA, USA). Dowex 100–200 mesh was sourced from Sigma‐Aldrich (Darmstadt, Germany), Media‐199 was obtained from Thermo Fisher Scientific and myo‐inositol and scintillation liquid from Perkin Elmer (Waltham, MA, USA).

### 2.2 HEK cell culture preparation and inositol phosphate (IP) accumulation assay

HEK 293‐T cell culturing and the IP accumulation assay were performed as described by Newton et al.^15^, ^16^ The IP assay measures the response of the cells to ligands that is a measure of the binding affinity and stimulation of intracellular signalling. This assay was applied to determine whether the novel ligand retained the same or improved activity as KP10.

HEK 293‐T cells were cultured and maintained using DMEM (10% FBS) media supplemented with Glutamax at 37°C, 5% CO_2_ and 95% relative humidity. HEK 293‐T cells were passaged at 90% confluency using Trypsin. HEK 293‐T cells were plated out into 24‐well plates at a density of 1 × 105 cells/well. Each well was pre‐coated with Matrigel at a 1:30 dilution to ensure adhesion to the well. KISS1R gene construct was transiently transfected into the HEK 293‐T cells at a concentration of 10 ng/μL of KISS1R using XtremeGene HP (1:2 ratio) DNA transfection reagent. Cells were incubated overnight at 37°C at 5% CO_2_.

For the IP assay, the HEK 293‐T cells were incubated with myoinositol‐[2‐3H] (3H‐myoinositol) (0.55 μCi/well) in IP Media (Medium‐199 supplemented with 1% FBS) at 37°C for 16 h. The IP medium was subsequently aspirated off and the cells incubated in Buffer I (140 mM NaCl, 4 mM KCl, 20 mM HEPES, 8 mM glucose, 0.1% SA, 1 mM MgCl2,1 mM CaCl2) supplemented with 10 mM LiCl2 for 30 min followed by a 1‐h incubation at 37°C with the ligands (unconjugated KP10 and DOTA‐KP10) in Buffer I. Following stimulation of the cells with the peptides, the cells were lysed for 1 h in 10 mM formic acid at 4°C. The radiolabelled IPs were separated using a Dowex 100–200 mesh resin ion‐exchange chromatography, and the radioactivity (cpm) was measured using liquid scintillation (Packard Tricarb 2100 TR liquid scintillation analyser TRI‐CARB 4910TR 110 V Liquid Scintillation Counter, Perkin‐Elmer, USA). The experiment was done in triplicate, and the three repeats were averaged. The results were analysed by doing sigmoidal regression to determine the EC 50 and then compared with their respective normalised ligand signalling assay.

The accumulation of the IPs measured for cells transfected with XtremeGene without KISS1R and treated with maximal stimulation of KP10 (1 × 10^−6^ M) was subtracted from the measured accumulation of cells stimulated with DOTA‐KP10 over a dose range (1 × 10^11^–1 × 10^−6^ M) that were transfected with KISS1R in the same assay.

### 2.3 Radiolabelling optimisation

The iThemba LABS ^68^Ge/^68^Ga‐generator (1.85 GBq, 1‐month‐old) was eluted by fractionated elution as described previously to reduce the concentration of contaminants that can influence radiolabelling efficiency.^17^ The ^68^Ga‐radiolabelling parameters for DOTA‐KP10 were based on a previously published procedure by us.^18^ In brief, eluate acidity, compound molarity, incubation time and temperature were evaluated. All labelling reactions were performed using a 1.0‐μg/μL peptide–chelator stock solutions (deionised water) comparing temperatures of 25°C (room temperature), 60 and 95°C. The reactions were monitored at 5‐min intervals over 20 min by determining the radiochemical purity as described in Section 2.5. Further radiolabelling was evaluated over a range of peptide–chelator concentrations (5–25 μM) and at pH ranging from 2.0 to 5.0 (note that the effect of pH was evaluated at suboptimal peptide concentration [5 μM] to ensure that any variations in the radiolabelling efficiency were clearly seen).

The preliminary evaluation of labelling of DOTA‐KP10 with lutetium‐177 was aimed at demonstrating the possibility of the development of a theranostic radiopharmaceutical. A method for ^177^Lu‐labelling suggested by Sinnes et al.^19^ was therefore followed and not further optimised. Non carrier added lutetium‐177 in 0.5 M HCl (100 MBq = ±0.1 nmol) was used, and DOTA is capable of complexing lutetium‐177 in molar ratios from 1:5 to 1:10 (lutetium‐177: chelator) for reproducible, quantitative labelling. Labelling was performed using 100 MBq lutetium‐177 (2–4 days old) added to 1 mL of DOTA‐KP10 (variable concentrations) and at a pH of 4.5 for 5 min. The temperature was kept constant at 95°C. After labelling was performed, the final product was buffered to a pH of 7 with 2.5 M sodium citrate for the stability study.

### 2.4 Product purification for in vivo analysis

To ensure that the product was >99% pure for further biological applications, [^68^Ga]Ga‐DOTA‐KP10 was purified as previously described.^17^ In brief, a C18 SepPak cartridge was conditioned with ethanol (>99.5%) and equilibrated with Millipore deionised water. The reaction product was loaded on the cartridge, washed and eluted with 1:1 (ethanol: saline). No more than 10% ethanol was used in the final preparation. In preparation for administration into animals, the ethanol contents were minimised by evaporation.

### 2.5 Radiochemical purity analysis

Instant thin‐layer chromatography (ITLC) was performed as described previously using silica‐gel TLC paper (ITLC‐SG). Each TLC was evaluated using two mobile phases: 0.1 M sodium citrate (pH 5.0) solution for determining the ionic (free) ^68^Ga‐species, and a mixture of 50:50 1 M ammonium acetate/methanol to determine the presence of colloidal ^68^Ga‐species.^17^, ^18^, ^20^ The radiolabelled product demonstrated an *R*_f_ value of 0.8–1.0 in the ammonium acetate/methanol system and 0.0–0.2 in the sodium citrate system.

High‐pressure liquid chromatography (HPLC) analysis was performed using an Agilent 1200 series HPLC (Wilmington, USA) instrument with a diode array UV detector. This system is coupled to a Gina Star Raytest (Strubenhardt, Germany) radio‐detector. A reverse‐phase X‐Bridge C18 HPLC column (Waters, Milford, USA) with a 3.5‐μm particle size, 3.0 × 100 mm was used for the radioanalysis in this investigation. The mobile phase consisted of solvent A (0.1% trifluoroacetic acid [TFA] in water) and solvent B (0.1% TFA in acetonitrile). The system was operated at a linear A‐B gradient with solvent B going from 5% to 95% over 20 min with a flow rate of 1 mL/min.

### 2.6 Log P octanol/phosphate buffer assay

The method published by Jain and co‐workers was used.^21^ Briefly, 100 μL (± 100 MBq) of the labelled [^68^Ga]Ga‐DOTA‐KP10 was dispersed in a 1‐mL phosphate‐buffered saline (PBS) and octanol mixture (0.9:1). The samples were vortexed (1 min) and then centrifuged (5 min, 6000 rpm). A sample (200 μL) from each phase was taken, and the activity was measured in the Hidex AMG automated gamma counter (Turku, Finland). The partition coefficient (log P) was expressed as the logarithm of the ratio of counts from the n‐octanol fraction versus that of the aqueous fraction.^21^, ^22^

### 2.7 Binding and stability studies

Bench‐top stability of [^68^Ga]Ga‐DOTA‐KP10 was performed by keeping the final formulated radiopharmaceutical (RCP > 95%) at room temperature (approximately 25°C) for a period of up to 3 h. This final formulation was buffered to a pH of 7 with PBS. Samples were collected at 30, 60 and 120 min and 3 h. ITLC and HPLC analyses were performed as described earlier. For the formulated [^177^Lu]Lu‐DOTA‐KP10 (RCP > 95%), the bench‐top stability analysis was performed at 24 h.

The red blood cell (RBC) binding assay was performed by the method described by Shi and co‐workers.^22^ In brief, radiolabelled [^68^Ga]Ga‐DOTA‐KP10 (740 kBq in 10 μL) was mixed with 100 μL of human blood drawn up in the presence of heparin. The mixture was incubated in a shaking incubator at 37°C. Samples (200 μL each) were collected at two time points, 10 and 30 min. Plasma and blood cells were separated by centrifugation (2000 rpm, 5 min). The plasma was removed, and the blood cells were washed twice with 500 μL of normal saline (0.9% NaCl) that was then added to the plasma fraction. The amount (%) of labelled compound that was bound to the RBC was determined by the ratio of distribution between the plasma and blood cell fraction.^22^

Stability studies were performed in triplicate using [^68^Ga]Ga‐DOTA‐KP10 spiked human serum, plasma or whole blood (samples were removed at 5, 60 and 120 min with incubation at 37°C). The [^68^Ga]Ga‐DOTA‐KP10 had a radiochemical purity of at least 95% purity (determined by HPLC analysis). The samples taken (200 μL) at the selected time points were treated with ice‐cold acetonitrile (500 μL) and thereafter filtered with a 0.22‐μm filter to remove all residual proteins in the extract. This sample was then analysed by the HPLC method as described under Section 2.2.4. In vitro stability study data from [^68^Ga]Ga‐DOTA‐KP10 was deemed as translatable for [^177^Lu]Lu‐DOTA‐KP10 also because preliminary investigations demonstrated the same phenomena is present for both.

The protein binding assay was done as described by Mdlophane et al.^18^ In brief, [^68^Ga]Ga‐DOTA‐KP10 was incubated for 0, 60 and 120 min in serum at 37°C. Proteins were precipitated in ice‐cold acetonitrile (500 μL) and centrifuged (5000 rpm, 10 min). The supernatant was removed, and the protein pellet was washed with acetonitrile (250 μL). The washed protein pellets and supernatants were measured in an automated gamma‐counter (Hidex, Finland).

### 2.8 In vivo evaluation

All procedures followed during the animal studies adhered to institutional and South African national guidelines for the care and use of laboratory animals (SANS10386:2021). Ethical approval for the study was obtained from the Research Animal Ethics Committee of North‐West University (NWU‐00560‐19‐S5) and the University of Pretoria (149/2019), South Africa. The animals were provided by the Department of Science and Technology/North‐West University Preclinical Drug Development Platform (PCDDP) Vivarium. The study was conducted at the Preclinical Imaging Facility (PCIF) of the Nuclear Medicine Research Infrastructure (NuMeRI), South Africa. Five healthy Sprague–Dawley rats (male, 6–8 weeks, ±200 g) were enrolled into the study. All animals had ad libitum access to a conventional rodent diet and water. Animals were maintained on a 12‐h artificial day/night cycle and supplied with corn cob bedding and nesting material for cage enrichment. Environmental conditions were kept stable in an individually ventilated cage system (Techniplast IVC, Milan, Italy). For imaging, rats were anaesthetised with an induction dose of 4% isoflurane in oxygen and maintained at 2% isoflurane in oxygen during the procedure. Image acquisition was done on a Mediso nanoPET/CT (Mediso, Hungary). The rats (*n* = 5) were administered intravenously with [^68^Ga]Ga‐DOTA‐KP10 (18.8 MBq ± 9.3 MBq) formulated in PBS to pH 7.0. The imaging protocol consisted of both a dynamic scan period (30–45 min) immediately following injection, as well as delayed image acquisition at 120–150 min post tracer injection. Dynamic images were regionally acquired: one bed position from the tip of the snout to below the diaphragm. Delayed whole‐body images were acquired over two bed positions. All list mode data acquired were corrected for scatter and randoms using the Nucline™ software (version:2.01.020.0000; MEDISO, Hungary) using an iterative reconstruction algorithm with computed tomography (CT) attenuation correction maps. Hybrid images were viewed and analysed using the InterviewFusion™ software (Mediso, Hungary). A qualitative visual analysis was done on all acquisitions, and blood standardized uptake value values were calculated.

### 2.9 Ex‐vivo biodistribution

After the final image was acquired (150 min postinjection), the animals were euthanised and ex vivo biodistribution was performed. Euthanasia was performed by an overdose of isoflurane (without waking up post‐scan) followed by decapitation. A quantitative analysis of the radioactivity biodistribution and uptake in various organs of the rats was conducted. Following micro‐PET/CT imaging, the anesthetised animals were euthanised by decapitation and blood was collected into pre‐weighed BD SST vacutainer tubes (thermos Fisher Scientific, Johannesburg, South Africa). The blood samples were centrifuged (6000 rpm, 5 min) to separate the serum from the blood cells. The blood and serum along with the other dissected tissues and organs (heart, liver, spleen, intestines, testes, stomach, lungs and brain) were weighed, and the radioactivity was measured in the automated gamma counter (Hidex AMG, Turku, Finland). The radioactivity per organ was expressed as the percentage injected dose per gram of the organ (% ID/g).

### 2.10 Statistical methods

All data analyses were performed with GraphPad Prism Software (Version 8.1 for Windows, GraphPad Software, San Diego, California, USA). If not stated otherwise, only data that have been executed at least in triplicate were considered relevant and were expressed as mean ± standard error mean (SEM). *p* < 0.05 was used to indicate statistical significance.

For the IP assay, the data were fitted using a nonlinear regression to determine Emax and EC50. Data collected from the ex vivo biodistribution were evaluated using the Kruskal–Wallis analysis. A chi‐squared that was less than the H‐statistic was used to indicate significant differences between the concentrations within regions of interest. Organs were measured for activity and expressed as the percentage injected dose per gram of the organ (%ID/g). A multipoint regression model (exponential fit) was used to estimate blood‐half life.

## 3 RESULTS

### 3.1 IP accumulation assay

**FIGURE 3:**
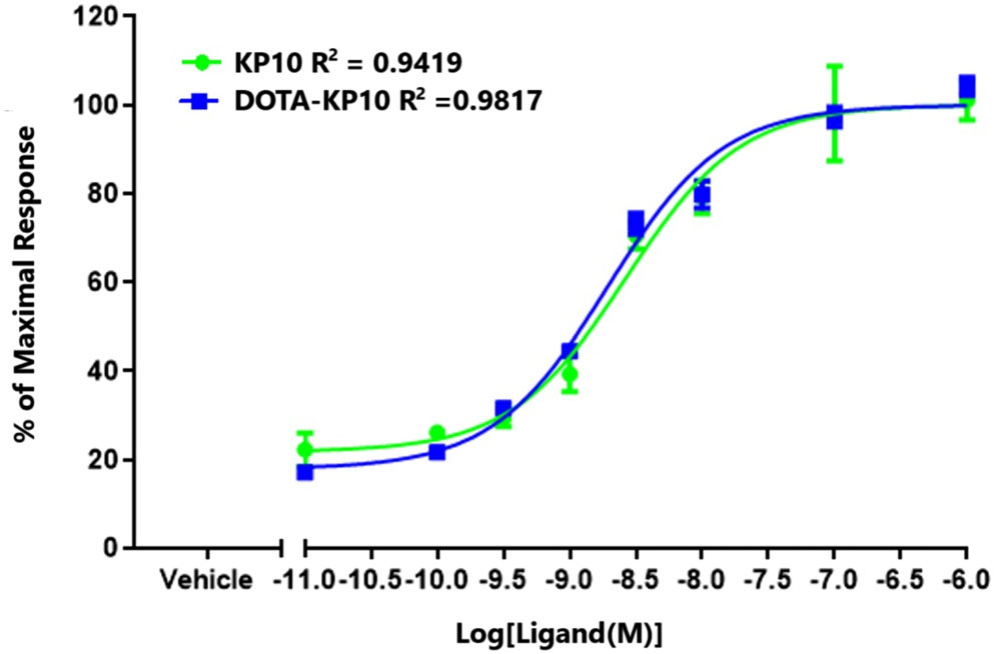
Stimulation of inositol phosphates accumulation in HEK293. cells expressing KISS1R by the DOTA‐KP10 ligand and native KP10. Data points were fitted by sigmoidal dose–response curves and are presented as a percentage of the maximal response of KP10 (set at 100%). Data are expressed as mean and SD from five independent assays. KP10 *R* ^2^ = 0.9419.

To determine if the functionalisation of KP10 with DOTA (for radiolabelling) was not influencing activity at the KISS1R receptor, the functional signalling assay of IP accumulation was utilised. This is a measure of both the ligand binding and receptor activation, thus an integrated measure of biological activity in vitro. The EC50s of KP10 and DOTA‐KP10 were determined as 1.87 × 10^−9^ M and 3.994 × 10^−9^ M respectively from four independent experiments (Table 1). The maximal response to DOTA‐KP10 was 97.8% of that of KP10 (Figure 3). There were no significant differences in these parameters, suggesting that DOTA‐KP10 could be developed as a theranostic for cancers expressing the kisspeptin receptor and for interrogating normal physiology.

**TABLE 1:** The effective concentrations that elicited 50% of the maximal response in HEK 293‐T cells expressing KISS1R when treated with KP10 and DOTA‐KP10. The data are represented as a mean with the standard error mean for a sample size of four. The values were not significantly different.

Ligand	EC_50_ Mean nM ± SEM (*N* = 4)
KP10	1.87 ± 0.095
DOTA‐KP‐10	3.99 0.075

Abbreviation: SEM, standard error mean.

### 3.2 Radiolabelling optimisation and radiochemical purity

**FIGURE 4:**
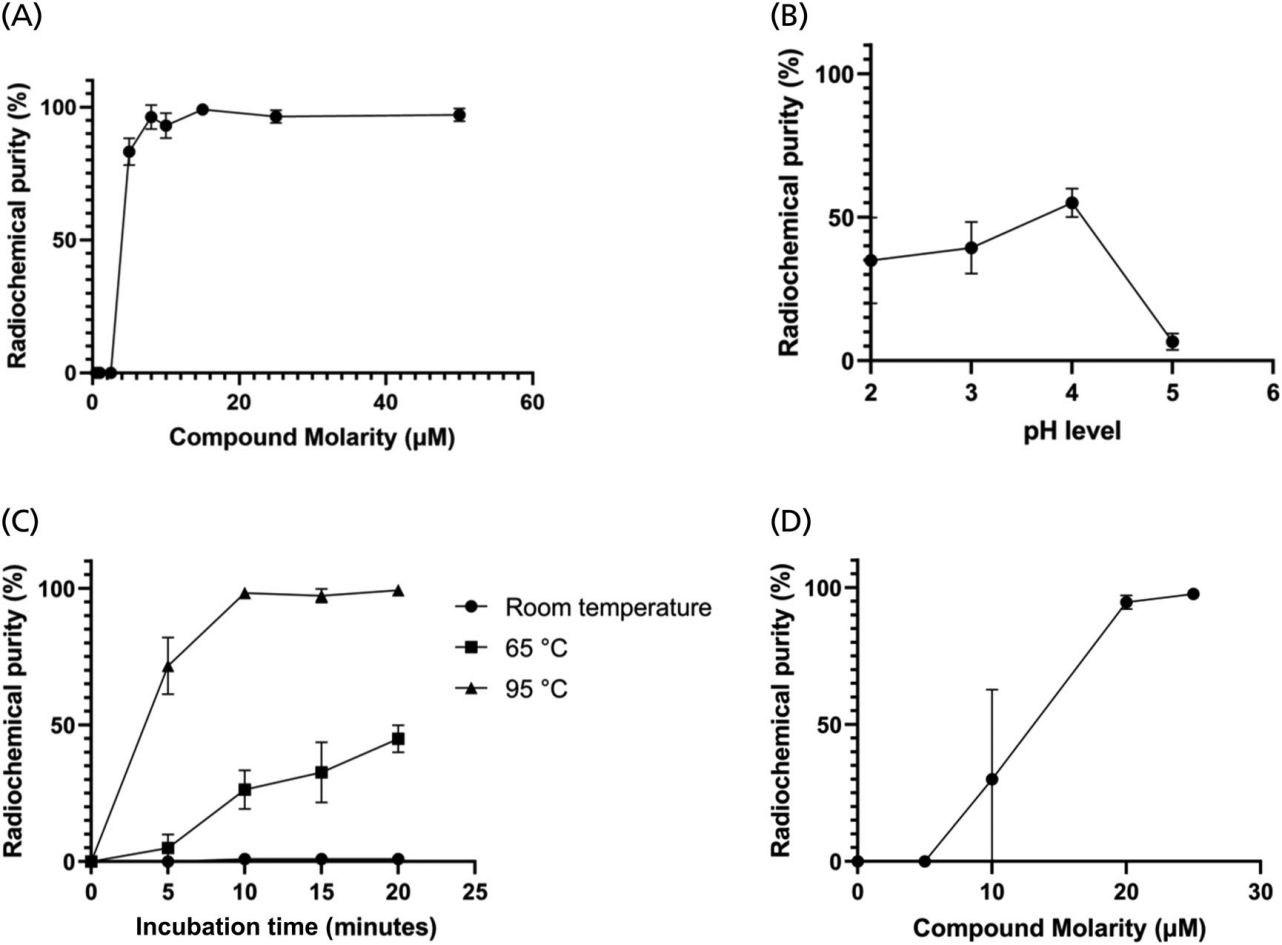
The effects of various parameters on the radiolabelling efficiency of gallium‐68 and compound molarity of lutetium‐177 with DOTA‐KP10. (A) The effect of compound molarity. (B) The effect of pH on ^68^Ga‐complexation. (C) The effects of temperature and incubation time on ^68^Ga‐product yield. (D) The effect of compound molarity on ^177^Lu‐labelling.

After a series of radiolabelling tests (Figure 4), a potential optimal radiosynthesis method identified for labelling of DOTA‐KP10 with gallium‐68 comprised a compound concentration of 10 μM incubated at a pH of 4.0 for 10 min at 95°C.

The optimisation of 68Ga‐labelling of DOTA‐KP10 was performed with different compound molarities ranging from 2.5 to 25 μM, and the results are shown in Figure 4. 68Ga‐radiolabelling became more quantitative at 7.5 μm (*N* = 3) with robust labelling results obtained using 10 μM of DOTA‐KP10 for the radiosynthesis. The pH range investigated was from 2.0 to 5.0 with labelling being considered optimal at a pH of 4.0 (Figure 4B). Colloidal gallium‐68, as well as free gallium‐68, was present at pH 3, and lower and other colloidal species become problematic at pH 5. Incubation times were evaluated from start of synthesis up to 20 min at various temperatures (room temperature, 60 and 95°C) (Figure 4C). Quantitative labelling was achieved at 95°C after 10 min of incubation time. For labelling of DOTA‐KP10 with lutetium‐177, only the compound molarity was investigated (Figure 4D).

**FIGURE 5:**
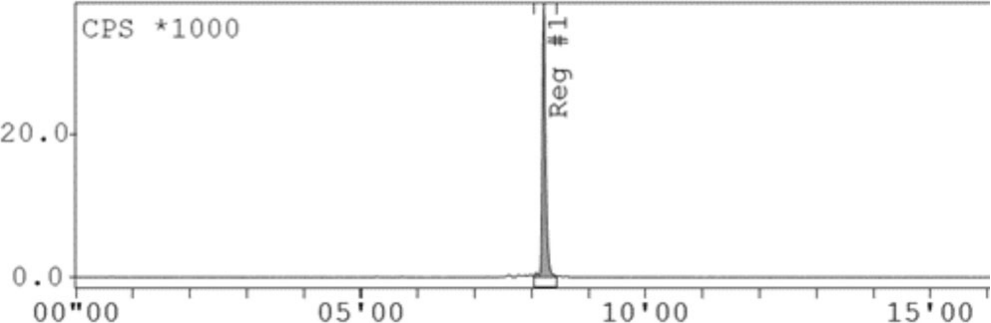
Radiochromatogram of a radiolabelling performed in optimal reaction conditions resulting in a radiochemical purity of more than 95%. Note the retention time of [^68^Ga]Ga‐DOTA‐KP10 at 8.2 min.

The apparent molar activity was 133 GBq/μmol, and the yield was decay corrected to an average of 82 ± 0.8% when C18 SepPak purification was performed. The analysis of radiochemical purity was performed by ITLC and confirmed with HPLC. For ^177^Lu‐labelling, only radio‐HPLC analysis was performed. A radiochromatogram with an optimal radiolabelling is shown in Figure 5. The rest of the chromatograms are provided in the (Supplement Data B).

For all animal studies, a well‐established product purification was performed using a C18 SepPak cartridge to achieve desalting, change of activity concentration and a radiochemical purity of more than 95%.^23^ For labelling of DOTA‐KP10 with lutetium‐177, only the compound molarity was investigated. High radiolabelling efficiency was achieved using a compound molarity of 20–25 μM. The apparent molar activity was 4 GBq/μmol, and the yield was 97 ± 2.3%.

### 3.3 Log P octanol/phosphate‐buffered assay

The accumulation of [^68^Ga]Ga‐DOTA‐KP10 in the aqueous phase was 99.1 ± 0.72% (*N* = 5) with a log *p* value of −2.0. This demonstrates that the compound is very water‐soluble that and likely to be excreted via the kidneys.

#### 3.3.1 Binding and stability studies

The red blood cell binding assay demonstrated that the binding of [^68^Ga]Ga‐KP10 was 6.72 ± 0.37% at 10 min and 6.35 ± 2.7% at 30 min. The [^68^Ga]Ga‐KP10 associated radioactivity bound to the plasma proteins increased gradually over time with 28.2 ± 1.35% after initiating the incubation, 46.4 ± 7.24% after 1 h and 67.99 ± 3.56% at 2 h of incubation (*N* = 3).

**FIGURE 6:**
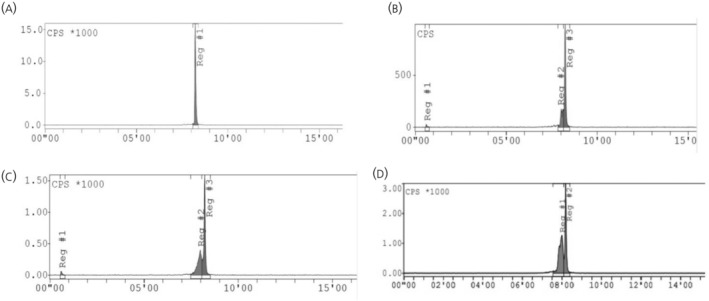
Example chromatograms of [^68^Ga]Ga‐DOTA‐KP10 demonstrating change in radiochemical purity: (A) control incubated without blood; (B) degradation at 5 min incubation with plasma; (C) degradation at 5 min incubation with serum; (D) degradation at 5 min incubation with whole blood.

The benchtop stability of [^68^Ga]Ga‐DOTA‐KP10 (*N* = 3) formulated in PBS over a 3‐h period showed no evidence of break down over 3 h. [^177^Lu]Lu‐DOTA‐KP10 (*N* = 3) was also stable over 24 h (Supplementary Data C). When incubated with whole blood, serum and plasma, [^68^Ga]Ga‐DOTA‐KP10 showed the formation of a degradation product on the radio‐chromatogram in the 5 min samples (Figure 6B–D). Stability was measured over time (5 min incubation, 1 and 2 h, *N* = 3). At 5 min, the change was already visible; no change in stability was observed over 2 h (Supplementary Data D).

### 3.4 In vivo evaluation and ex‐vivo biodistribution

**FIGURE 7:**
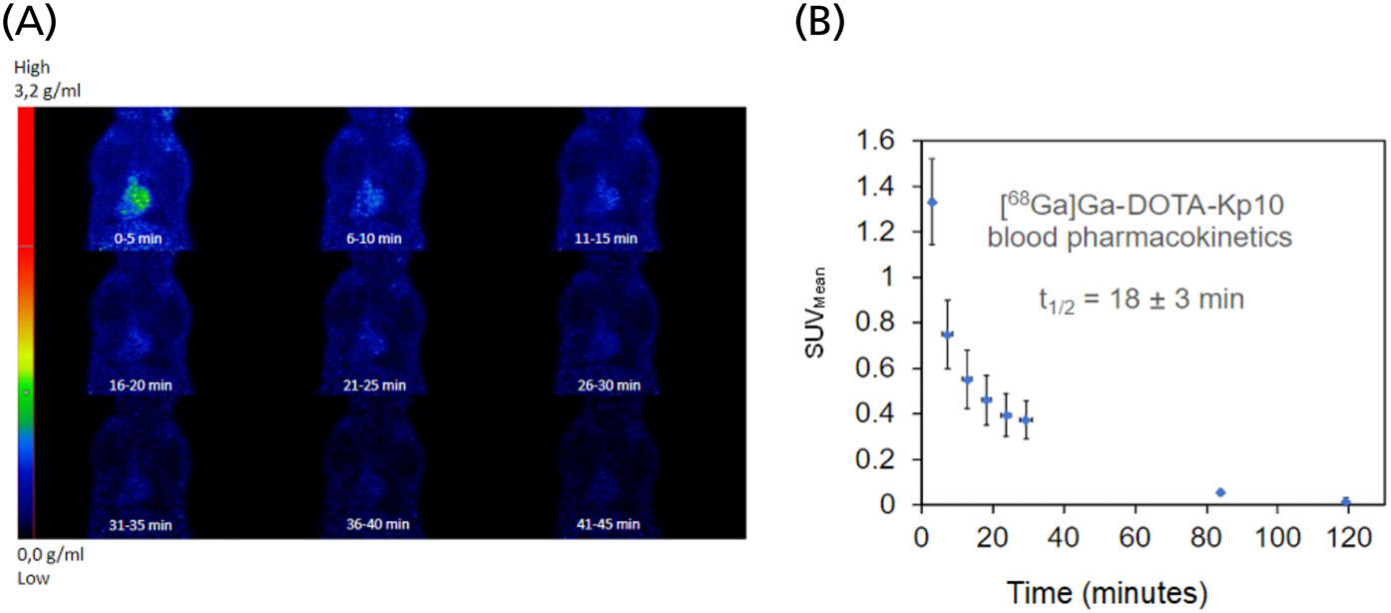
Dynamic micro‐PET/CT imaging in different coronal cross sections (A) showing a gradual decrease in blood pool activity over 34 min; the image‐guided (CT based region of interest of the myocardium, *N* = 4). SUV analysis was performed to draw the time‐activity curve (B) to allow for calculation of the physiological half‐life of [^68^Ga]Ga‐DOTA‐KP10 (*R* ^2^ = 0.895). CT, computerised tomography; PET, positron emission tomography; SUV, standardized uptake value.

**FIGURE 8:**
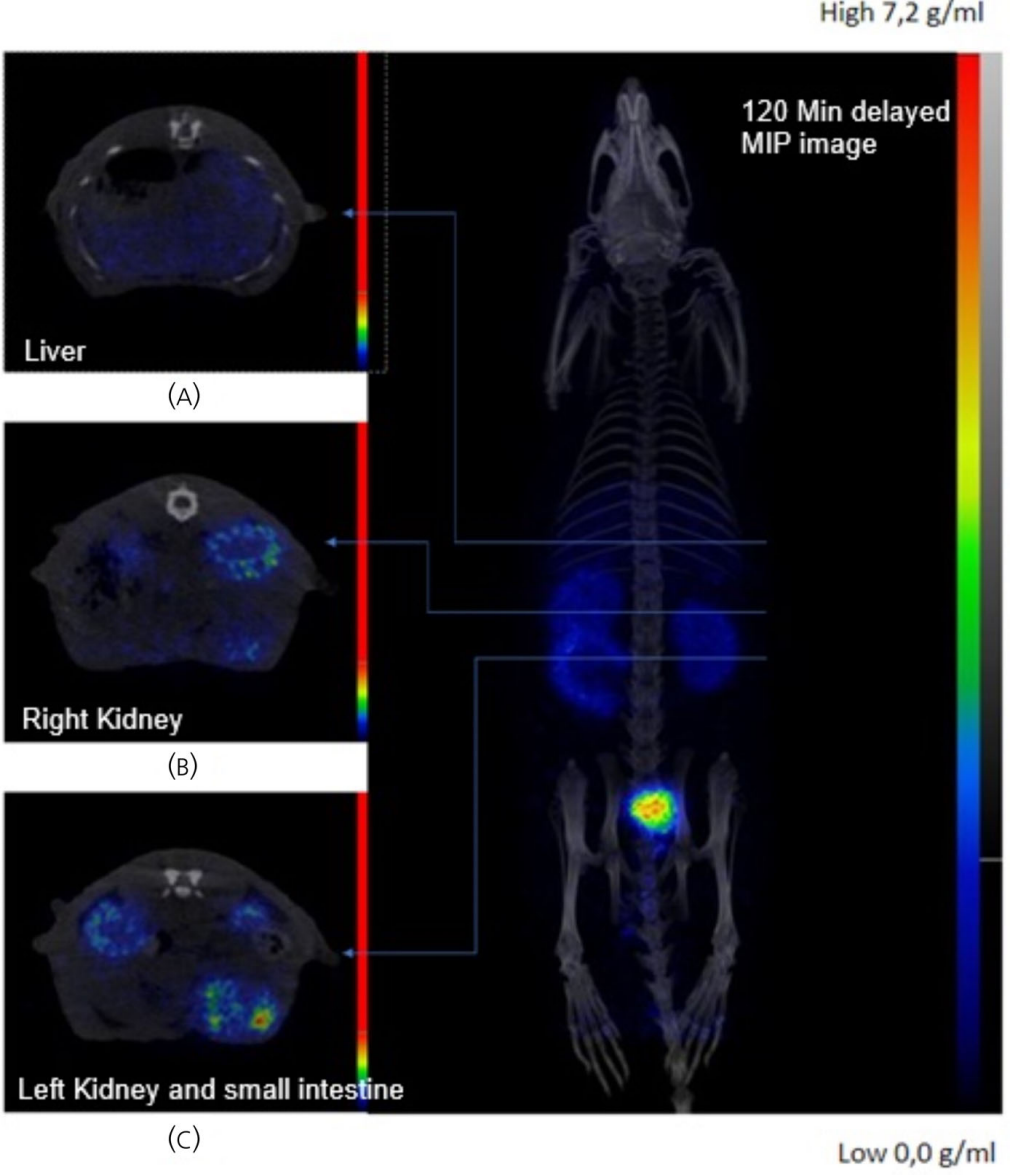
An example of a full body image (prone) in maximum intensity projection (right panel) from a 120‐min delayed micro‐PET/CT acquisition (right) with cross‐sectional micro‐PET/CT image slices (left panel) demonstrating noticeable radioactivity in (A) liver, (B) right kidney, (C) left kidney and small intestine. CT, computerised tomography; PET, positron emission tomography.

**FIGURE 9:**
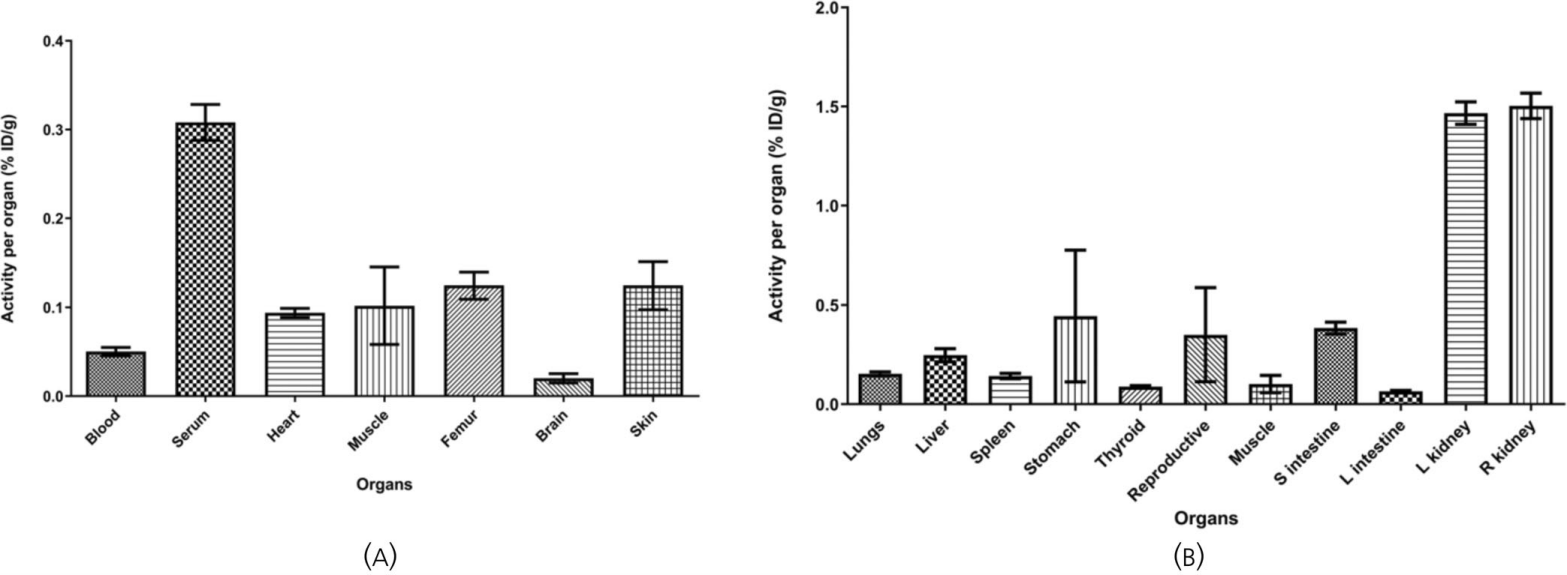
[^68^Ga]Ga‐DOTA‐KP10 biodistribution derived from ex vivo gamma counting of dissected organs at 150 min postinjection. Results are presented as % injected dose per gram. Data are expressed as mean and SD (*N* = 5). On the right graph (A), major organs are indicated, and on the left graph, (B) excretory organs are provided. Note the low uptake by the brain.

Dynamic uptake studies with micro‐PET/CT after [^68^Ga]Ga‐DOTA‐KP10 administration on bed (injection performed directly after the start of image acquisition) indicated that the background clearance of this radiopharmaceutical was rapid. The blood half‐life was 18 ± 3 min (Figure 7). At 120 min, radioactivity was still excreted as visualised by an elevated bladder and kidney signal (Figure 8). Based on the visual qualitative data, it is unlikely that the radiopharmaceutical will be persistent in the kidneys. Ex vivo organ radioactivity was analysed and used for %ID/g calculation (Figure 9); no organs accumulated the radiopharmaceutical significantly (<0.5% ID/g, except for kidneys [1.5 ± 0.2% ID/g]). Activity presence in the kidneys was maintained at 150 min as well as noticeable activity in the stomach.

## 4 DISCUSSION

The original discovery of differential expression of KISS1R in metastatic and nonmetastatic melanoma cell lines sparked extensive studies on the bio‐oncology of KP and KISS1R in numerous cancers that reported a diversity of effects; many of which were contradictory.^1^, ^2^, ^3^ To further understand the role of the KP/ KISS1R involvement in normal physiology and pathologies, a radiolabelled KP has potential as a biomarker to visualise KISS1R expression in vivo to follow disease progression and monitor therapeutic response with PET/CT imaging. Moreover, it provides the opportunity to clarify and resolve the role of KP in cancer progression. For example, in triple‐negative breast cancer and hepatocellular carcinoma, [^68^Ga]Ga‐DOTA‐KP10 could be predictive of a more aggressive disease, and as a theranostic, [^177^Lu]Lu‐DOTA‐KP10 could open unexplored avenues for radioligand therapy.

Our current investigation provides initial steps on the feasibility towards developing such a radiopharmaceutical as there have not been studies utilising KP10 derivatives for intravenous administration and in vivo imaging or radiotherapy. While this article was in preparation, another group has also embarked on investigating this target with a NODAGA‐KiSS‐54 radiolabelled with gallium‐68^24^ as a diagnostic analogue. Their study also demonstrated potential application of kisspeptin in diagnostic oncology. Our study shows that a much smaller peptide sequence with full biological activity, namely KP10, can be used to develop novel radiopharmaceutical analogues requiring less complex and expensive procedures. Our study presents robust data demonstrating that KP‐10 conjugated to DOTA via the NH2‐terminus retains full biological activity. This concurs with SAR studies, which demonstrated that structural modification at the NH2‐terminus of KP10 did not impair receptor binding and activation.^11^, ^12^, ^13^, ^14^ We chose to use a functional assay of IP signalling rather than receptor binding in the development because it is more indicative for evaluating the in vivo activity. The functional assay is a product of both the binding affinity and receptor activation. Moreover, binding and activation of the receptor leads to internalisation of an agonist that is desirable for a radiochemical therapeutic.

Our investigation also reported robust radiosynthesis procedures for the complexation of gallium‐68 and in addition lutetium‐177 that proceeded well in line with other PRRT peptides. Both gallium‐68 and lutetium‐177 radiolabelling optimisations resulted in products with a radiochemical purity higher than 95%, meeting the release criteria for molar activity, activity concentration and radiochemical yield and total yield/radiosynthesis. The optimal radiolabelling molarity for lutetium‐177 was >20 μM when employing similar conditions as those used for ^68^Ga‐labelling (10 uM, pH 4.0, 95°C, 10 min). Encouragingly, no formation of radiolysis products or other impurities was present during stability assays of the formulated products (pH 7) at room temperature (approximately 25°C). These formulations are suitable for intravenous injections. These [^68^Ga]Ga‐DOTA‐KP10 samples were stable for a sufficient period to dispense and analyse multiple doses (2 h), and [^177^Lu]Lu‐DOTA‐KP10 was stable for at least 24‐h duration.

[^68^Ga]Ga‐DOTA‐KP10 protein binding could influence renal clearance as after 1 h, this was determined to be 46.4 ± 1.35% and increases over time. The physiological half‐life of 13 min in healthy rodents is acceptable for further investigations. It demonstrates a quick clearance of the non‐targeted radiopharmaceutical. Because Kisspeptin is expressed in the hypothalamus as a major regulator of the reproductive axis, we investigated if any uptake was demonstrable in the micro‐PET/CT images. No uptake was observed that is probably due to the very small number of GnRH neurones expressing the kisspeptin receptor and rapid degradation and metabolic clearance. We have designed protease‐resistant DOTA‐kisspeptin molecules along the lines of that described by literature^23^ that we will be testing.

The amount of the radiopharmaceutical associated with red blood cells was demonstrated to be negligible. There was a gradual but moderate formation of an additional radioactive peak preceding the main product peak detected by radio‐HPLC analysis of [^68^Ga]Ga‐DOTA‐KP10 incubated in human serum, plasma and whole blood. This is likely a proteolytic product as KP10 is known to be degraded by a number of proteases.^23^ In a study by Takeda Pharmaceuticals, the COOH‐terminal peptide bond between Arg9‐Phe10 was degradable by trypsin‐like proteases and the Tyr1‐Asn2 amino acid pair underwent NH2‐terminal degradation by aminopeptidases.^23^ The intra‐peptidyl cleavage sites at Gly7 may be vulnerable towards chymotrypsin‐like proteases, neutral endopeptidases and matrix metalloproteases.^23^ Additionally, asparagine residues (here Asn2) have been shown to undergo spontaneous rearrangement in aqueous environments.^23^, ^25^, ^26^, ^27^ Hence, one or more of these amino acids (Tyr1, Asn2, Gly7 and Arg9) may account for the serum instability. Instability can be addressed by the substitution of the vulnerable amino acids with D‐amino acids and unnatural synthetic amino acids—an approach that is extensively employed for biological peptides. This has already been established in the structure of TAK 448.^23^ Conjugation of this compound with a DOTA‐derivatised NH2‐terminus and other enhanced‐activity KP10 agonists is likely to deliver increased activity and reduced degradation.

This study is the first to design and characterise DOTA labelled KP‐10 and undertake the in vivo characteristics and pharmacokinetics of radiolabelled KP10 using dynamic micro‐PET/CT for its visualisation in healthy rats. These results demonstrated a decrease in blood pool activity of [^68^Ga]Ga‐DOTA‐KP10 primarily through renal clearance with no visible, unexpected off‐target organ accumulation of activity. From this exploratory study design, a sixfold to eightfold serum:blood ratio was established from ex vivo sample gamma counting. Gamma analysis indicated that the radiopharmaceutical may also show signs of kidney retention, which in the light of cancer theranostics, should be further investigated. The micro‐PET/CT image analysis showed uniform distribution of activity in vasculature and soft tissue over the 45‐min dynamic scan period with no noticeable accumulation in the brain or lungs. Delayed, [^68^Ga]Ga‐DOTA‐KP10 micro‐PET/CT images were acquired (at 120–145 min) for a whole‐body representation of tracer biodistribution, thereby showing no vascular retention, lung or brain uptake with clearance through the expected excretory routes (renal > hepatobiliary). These observations were confirmed with the ex vivo organ distribution.

We have demonstrated that DOTA‐kisspeptin retains full efficacy and potency when compared with the parent compound but is rapidly degraded in vivo and this presumably accounts for a lack of signal in the hypothalamus. However, the synthesis of DOTA‐kisspeptin analogues is straightforward so we are synthesising protease‐resistant analogues along the lines. Explored by Takeda researchers that also display higher affinity and lower metabolic clearance.^23^ Good property compounds will be evaluated in pan‐tumour xenografts for diagnostic capabilities (with gallium‐68) and with lutetium‐177 for therapeutic efficacy.

## 5 CONCLUSION

We report on the design and characterisation of a novel DOTA‐KP‐10 compound that retains full biological activity as measured by inositol signalling in cells expressing KISS1R. We further established conditions for efficient high‐level labelling with gallium‐68 and lutetium‐177 that establishes a point of departure for the development as an oncological diagnostic and therapeutic respectively. The labelled compounds may also be of value in identifying novel issues regulated by kisspeptin in normal physiology. There are limitations of the current compounds presented here, mainly with regard to the possible rapid degradation through the multiple proteolytic sites in KP‐10. To overcome this, we have synthesised a high affinity and proteolytic‐resistant DOTA‐KP‐10 that we are characterising.

## AUTHOR CONTRIBUTIONS

**Janke Kleynhans:** Investigation; writing – original draft; visualization; methodology; formal analysis; data curation. **Robert Reeve:** Investigation; methodology; visualization; validation; data curation. **Cathryn H. S. Driver:** Conceptualization; investigation; validation; visualization; writing – review and editing; supervision. **Biljana Marjanovic‐Painter:** Formal analysis; methodology; validation; writing – review and editing. **Mike Sathekge:** Supervision; resources; project administration; writing – review and editing; conceptualization. **Jan Rijn Zeevaart:** Conceptualization; funding acquisition; visualization; formal analysis; project administration. **Thomas Ebenhan:** Project administration; resources; writing – review and editing; investigation. **Robert P. Millar:** Writing – review and editing; writing – original draft; investigation; conceptualization; methodology; supervision; resources; project administration.

## FUNDING INFORMATION

RPM acknowledges grant support from the National Research Foundation and the Medical Research Council (South Africa).

### PEER REVIEW

The peer review history for this article is available at https://www.webofscience.com/api/gateway/wos/peer‐review/10.1111/jne.13487.

## Supporting information


Data S1.

## Data Availability

The data that support the findings of this study are available from the corresponding author upon reasonable request.
