# Supplementary material for: Synthesis and characterisation of DOTA‐kisspeptin‐10 as a potential gallium‐68/lutetium‐177 pan‐tumour radiopharmaceutical
Source: J Neuroendocrinol. 2025 Jan 7;37(3):e13487. doi: 10.1111/jne.13487 (PMC11919473; doi:10.1111/jne.13487)
Supplement: Supplementary file 1 — Data S1. [file JNE-37-e13487-s001.docx]

Supplementary materials

**Synthesis and characterisation of DOTA-Kisspeptin-10 as a potential gallium-68/lutetium-177 pan-tumour radiopharmaceutical**

**Supplementary Data A**


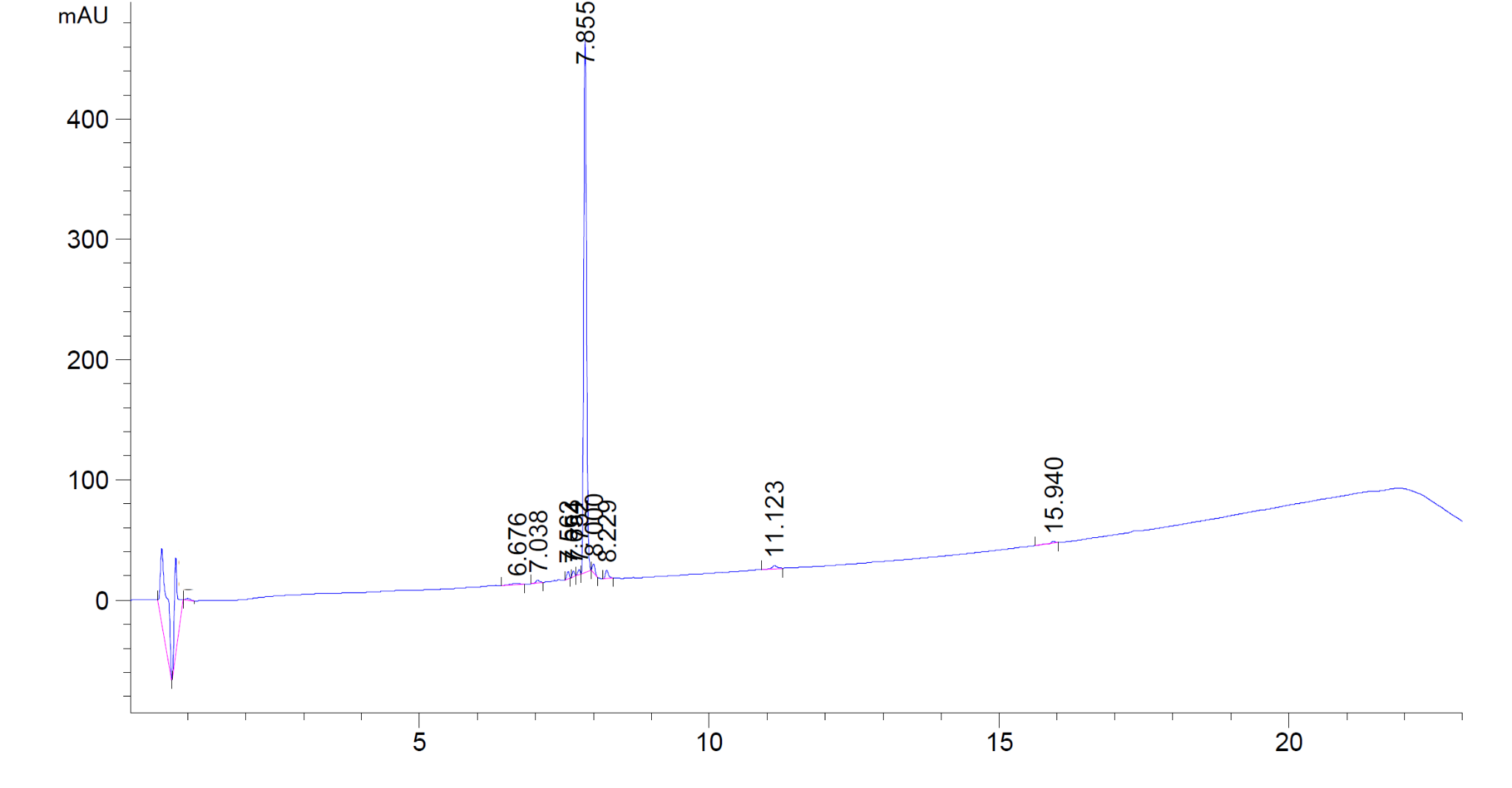


**Supplementary Data B**

UV determination of retention time of unlabelled DOTA-KP-10 with the signal positioned at 7.8 minutes and the method run for 20 minutes. This was performed with unlabelled DOTA-KP10


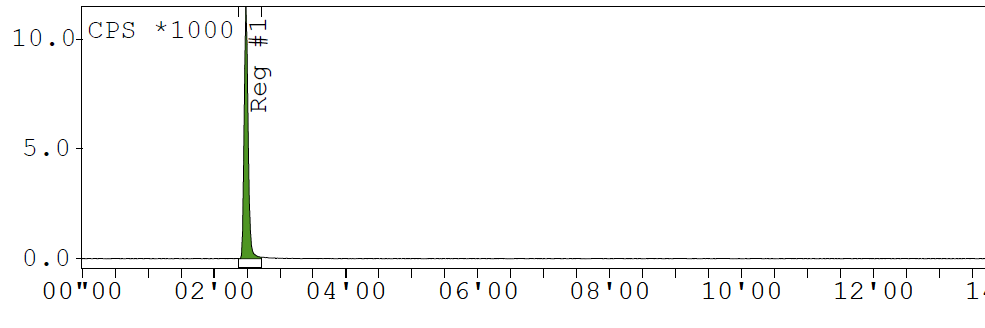


Radiochemical purity of 0% with only free gallium-68 present in the sample.


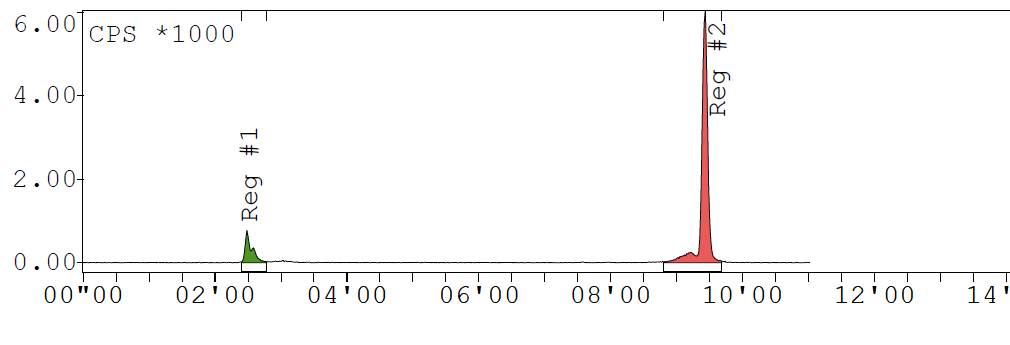


Radiochromatogram of radiolabelling in suboptimal reaction conditions resulting in a radiochemical purity of less than 95%.


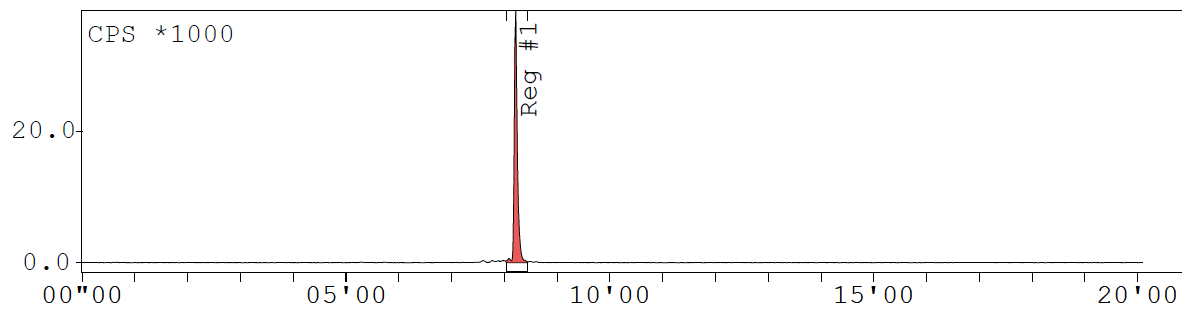


Radiochromatogram of a radiolabelling performed in optimal reaction conditions resulting in a radiochemical purity of more than 95%. Note the retention time of [^68^Ga]Ga-DOTA-KP10 at 8.2 minutes.


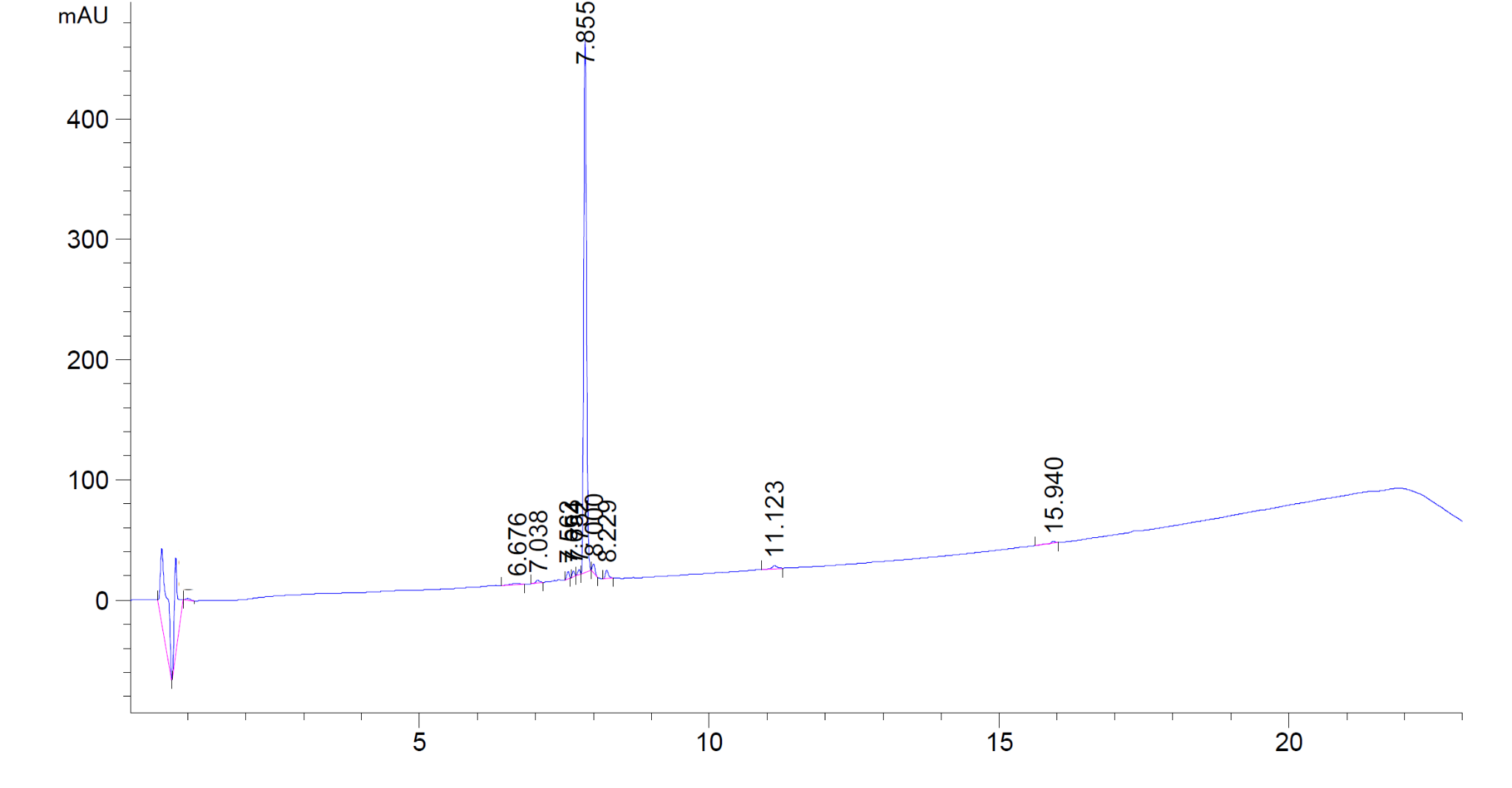


UV determination of retention time of unlabelled DOTA-KP-10 with the signal positioned at 7.8 minutes and the method run for 20 minutes. This was performed with unlabelled DOTA-KP10


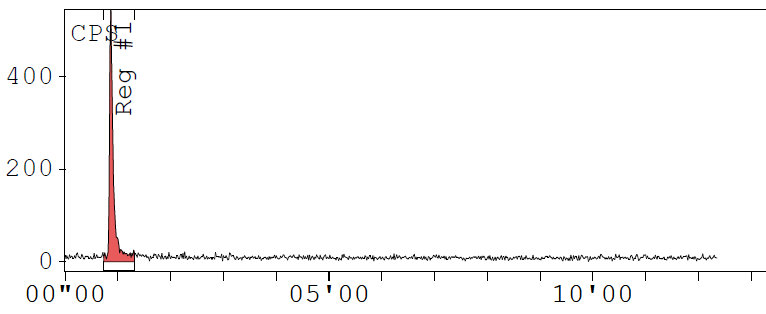


Radiochemical purity of 0% with only free lutetium-177 present in the sample.


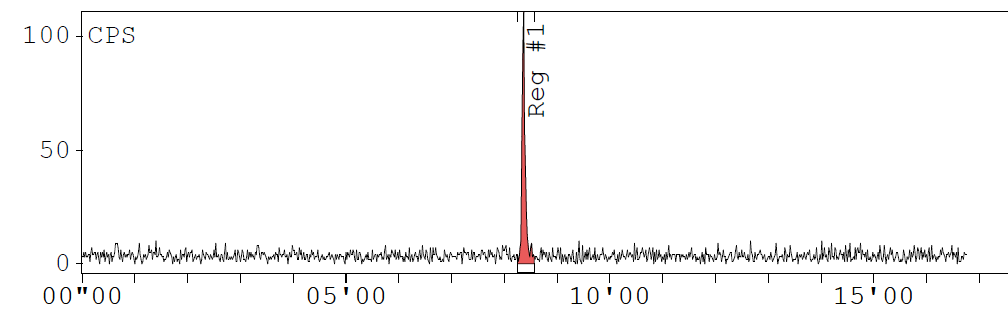


Radiochromatogram of a radiolabelling performed in optimal reaction conditions resulting in a radiochemical purity of more than 95%. Note the retention time of [^177^Lu]Lu-DOTA-KP10 at 8.2 minutes.

**Supplementary Data C**

| 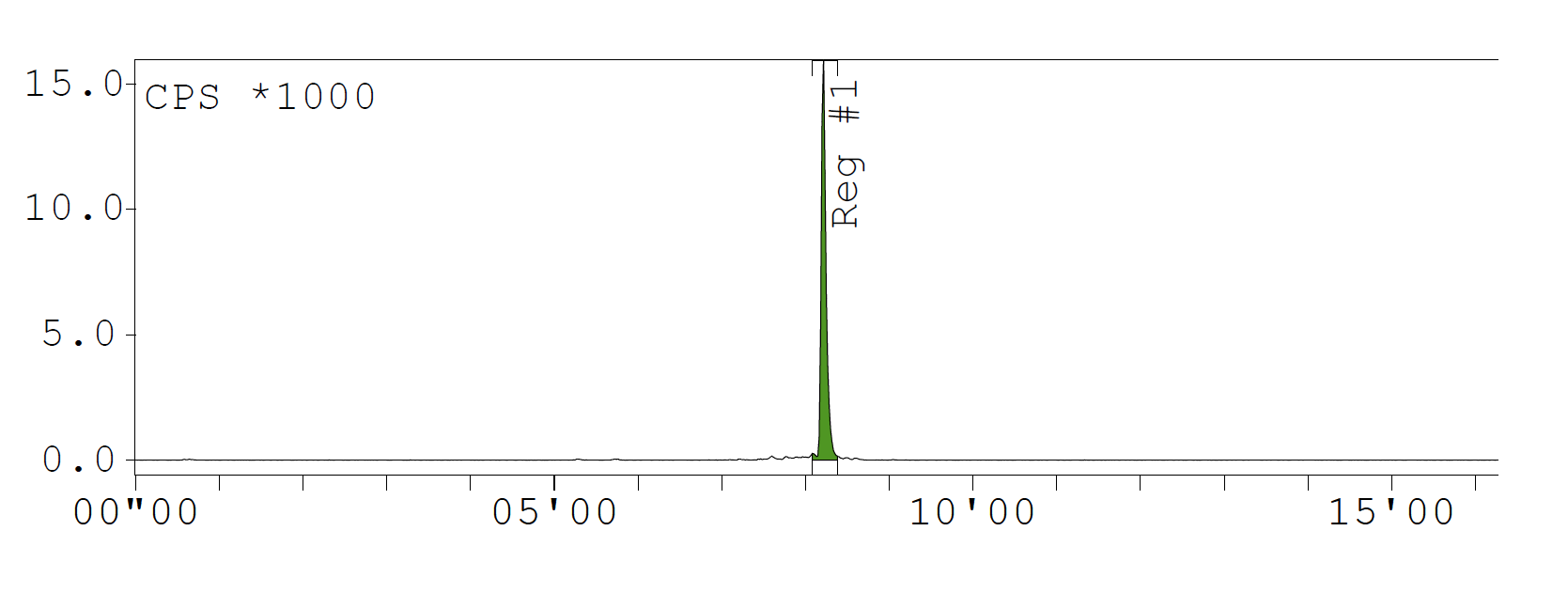 | 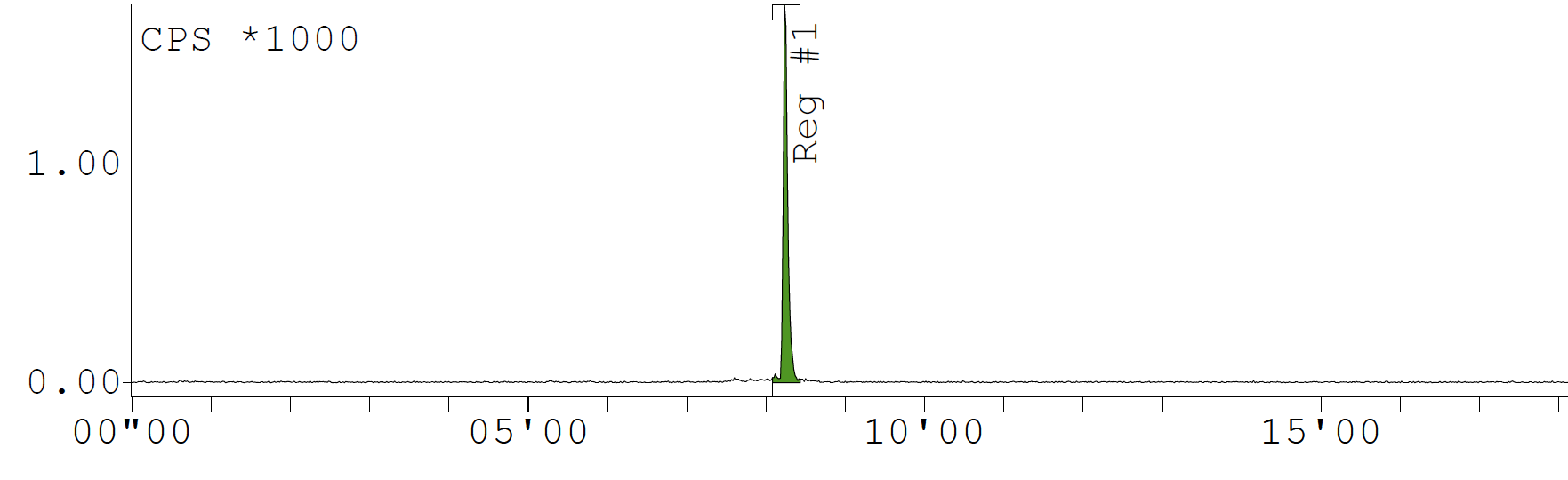 |
| --- | --- |

**[^68^Ga]Ga-DOTA-KP-10 Time 0 and time 3 hours bench -top Stability**

| 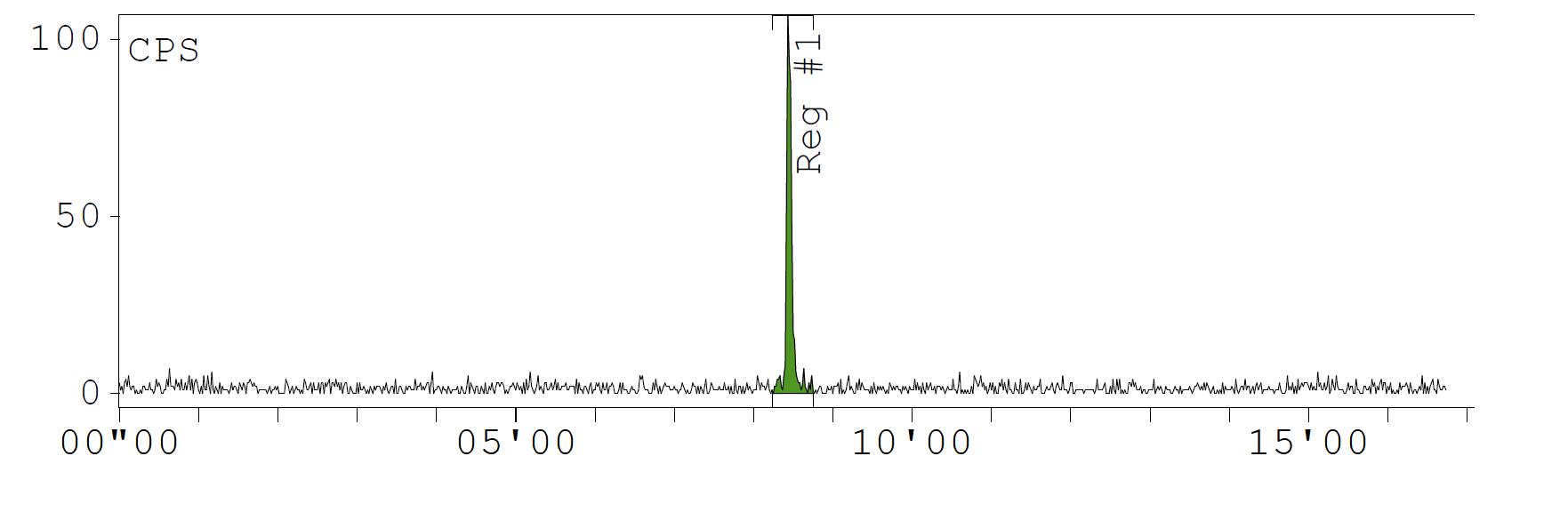 | 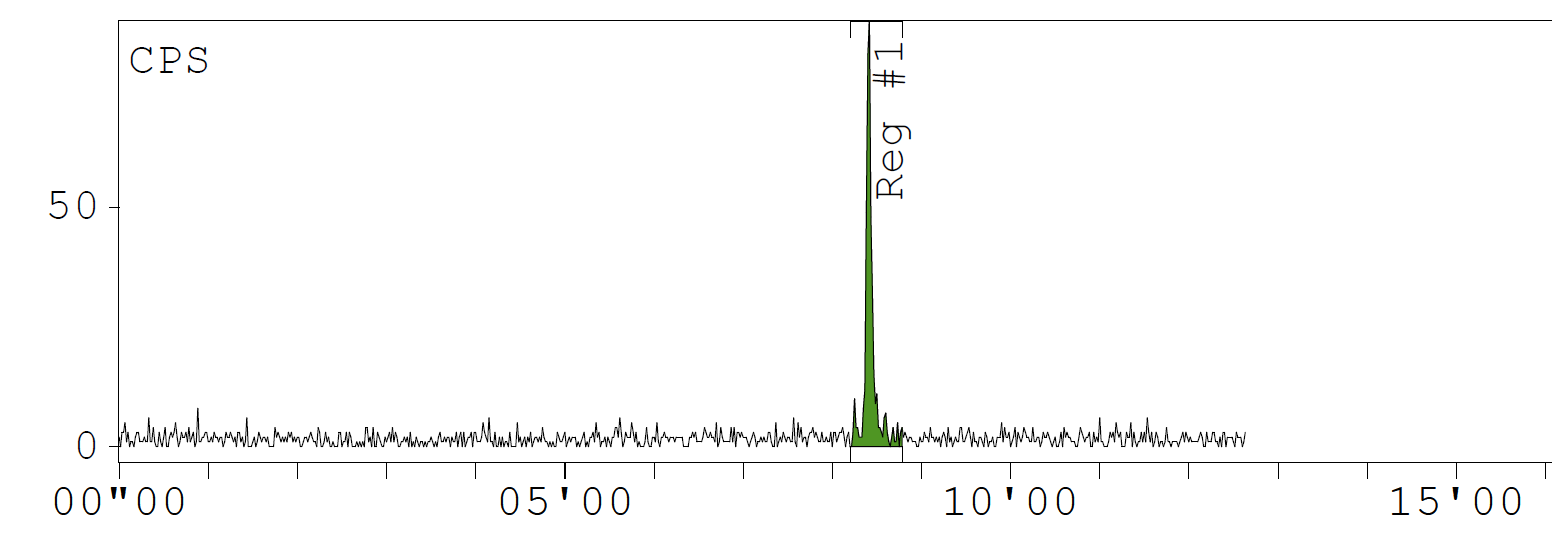 |
| --- | --- |

**[^177^Lu]Lu-DOTA-KP-10 Time 0 and Time 24 hours bench-top stability**

| Supplementary Data D | | |
| --- | --- | --- |
| 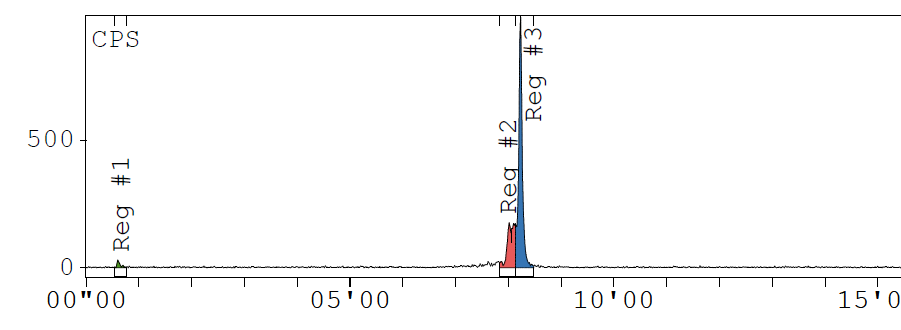 | 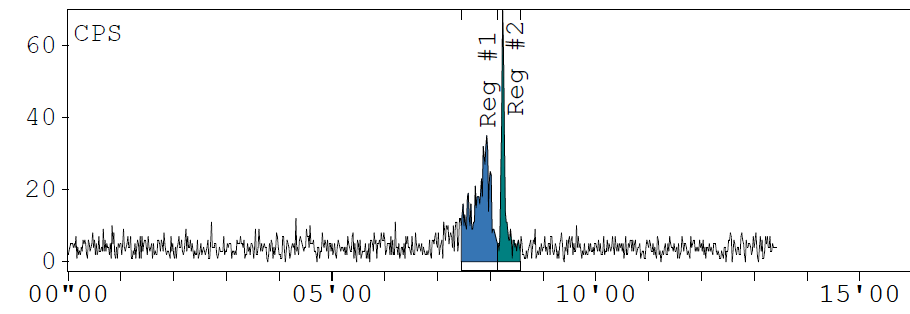 | 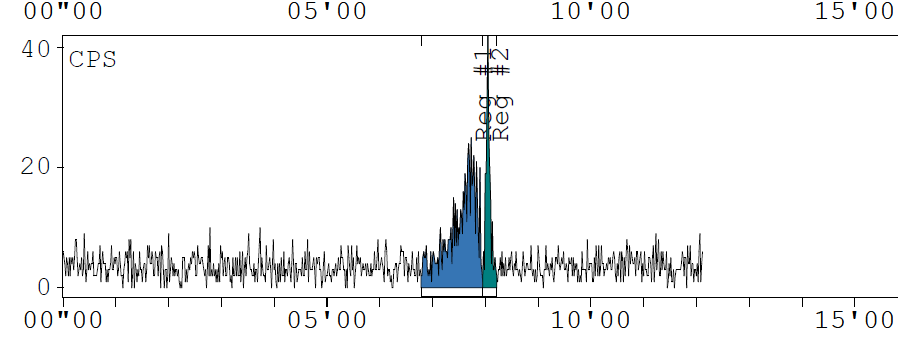 |
| **Plasma stability [^68^Ga]Ga-DOTA-KP10 time 5 min** | **Plasma stability [^68^Ga]Ga-DOTA-KP10 time 60 min** | **Plasma stability [^68^Ga]Ga-DOTA-KP10 time 120 min** |
| 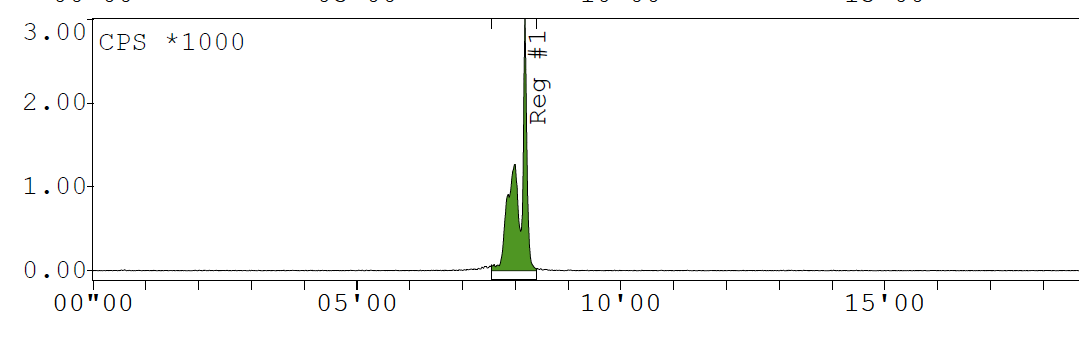 | 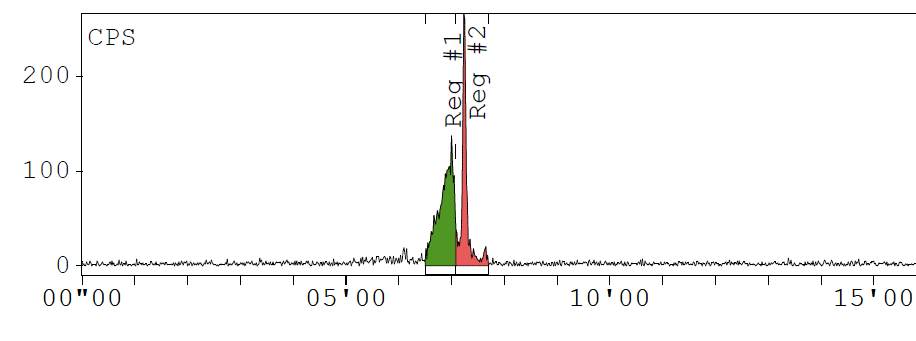 | 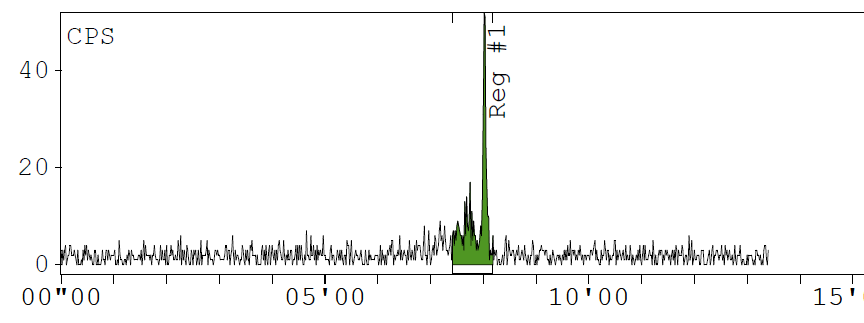 |
| **Whole blood [^68^Ga]Ga-DOTA-KP10 time 5 min** | **Whole blood [^68^Ga]Ga-DOTA-KP10 time 60 min** | **Whole blood [^68^Ga]Ga-DOTA-KP10 time 120 min** |
| 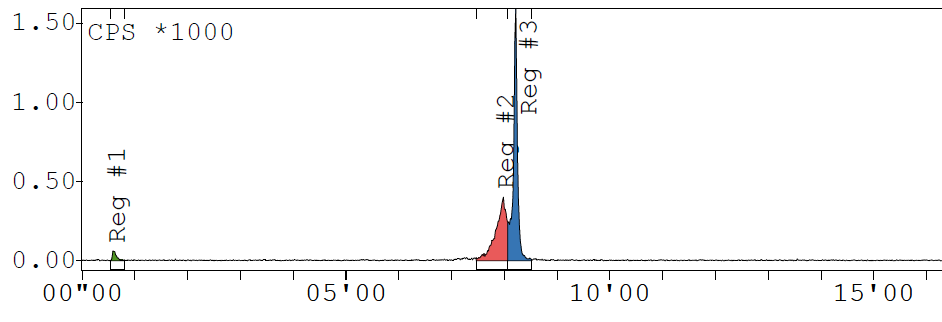 | 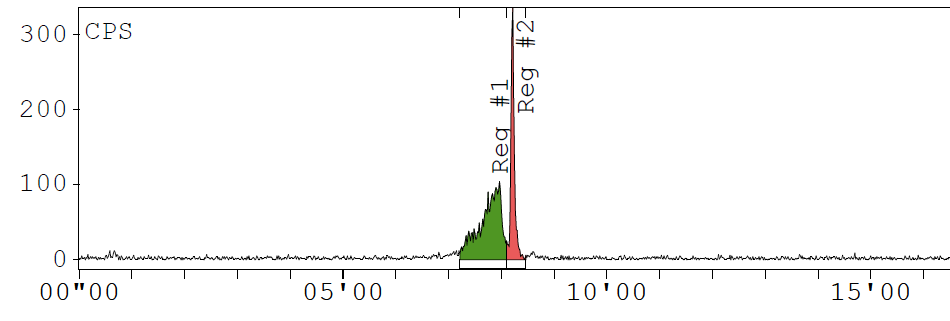 | 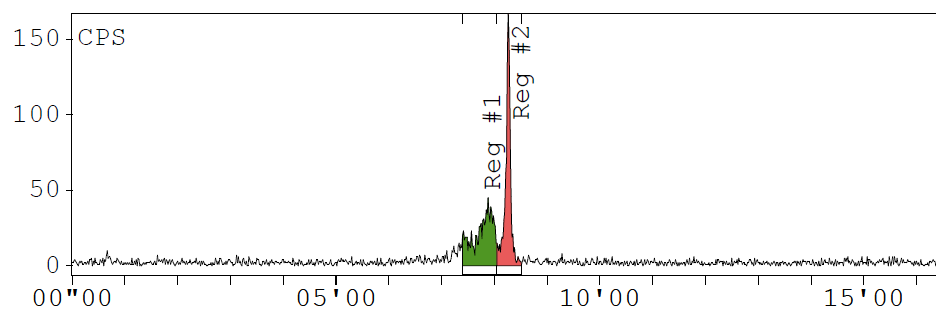 |
| **Serum stability [^68^Ga]Ga-DOTA-KP10 time 5 min** | **Serum stability [^68^Ga]Ga-DOTA-KP10 time 60 min** | **Serum stability [^68^Ga]Ga-DOTA-KP10 time 120 min** |
